# Mortality and continuity of care – Definitions matter! A cohort study in diabetics

**DOI:** 10.1371/journal.pone.0191386

**Published:** 2018-01-19

**Authors:** Angelika Geroldinger, Simone Katja Sauter, Georg Heinze, Gottfried Endel, Wolfgang Dorda, Georg Duftschmid

**Affiliations:** 1 Center for Medical Statistics, Informatics and Intelligent Systems, Medical University of Vienna, Vienna, Austria; 2 Main Association of Austrian Social Security Organizations, Vienna, Austria; University of Alberta, CANADA

## Abstract

**Objective:**

To demonstrate that when investigating the relevance of continuity of care for patient outcomes, different definitions can lead to contradicting results.

**Methods:**

We used claims data from the regional public health insurer of Lower Austria covering the period from 2008 to 2011. The study sample included subjects with repeated dispensings of anti-diabetic drugs. The continuity of care index was calculated firstly based on a patient’s contacts with general practitioners (primary COCI) and secondly based on contacts at all medical disciplines (total COCI). The association of the two continuity of care measures with mortality was assessed in separate univariable and multivariable Cox regression models.

**Results:**

Our study sample consisted of 51,717 patients with a median observation time of 3.65 years. The data showed that a high total COCI was associated with increased mortality, while there was no association between primary COCI and mortality.

**Conclusions:**

Measures of continuity of care are highly sensitive to the type of medical disciplines taken into account. The continuity of care index calculated from contacts at all medical disciplines might measure diversity rather than continuity of care.

## Introduction

Continuity of care (COC) and its association with different outcomes, such as mortality, hospitalization or healthcare costs, have been subject of numerous studies [1–6]. It might seem intuitive to define COC as how a patient experiences care over time as coherent and linked [7]. The more delicate question is how to quantify COC. A systematic review identified 32 different indices used to measure COC [8]. A popular choice are measures based on the frequency of contacts or on the distribution of care among multiple providers [7, 9]. These indices can often be calculated using administrative data sources. Typical examples are the Sequential Continuity index [10], the Usual Provider of Care index (UPC) [11] or the Continuity of Care index (COCI) [12]. According to the classification proposed by Jee and Cabana [8], the UPC, which is given by the proportion of visits made to the ‘usual provider’, is considered a density measure. We will later focus on the COCI, which is a measure of dispersion, i.e. it takes into account the distribution of visits among distinct providers. For the sake of completeness, we should mention that COC can also be assessed by patient surveys instead of measures derived from the pattern of contacts. While surveys might capture the patient’s perspective better, they are usually more resource and time intensive and carry the risk of selection bias due to varying response rates.

Deriving proxies for COC from the pattern of patient contacts with healthcare providers, one does not only have to make the choice of index. Another important question is which types of providers to consider. Two obvious options are to either use only contacts with general practitioners (GPs) in order to measure continuity of *primary* care, or, alternatively, to take into account contacts at all medical disciplines. The present study aims to illustrate the significance of the choice of the types of providers taken into account by demonstrating that it can have considerable impact on the analysis and its conclusions. To this end, we examined the association of COC with mortality in the diabetic population of Lower Austria using healthcare claims data. We calculated two versions of the COCI [12], based on contacts with GPs only and based on contacts at all medical disciplines, and contrasted their association with mortality. Finally, the results were critically reviewed with regard to interpretability. This paper extends an earlier version that was submitted to HEC2016 as an abstract [13].

## Methods

### Data source

We made use of a research data base maintained by the Main Association of Austrian Social Security Institutions, which contains de-identified reimbursement data for outpatient services of the regional health insurance carrier of Lower Austria (Niederösterreichische Gebietskrankenkasse, NÖGKK) from the years 2008 to 2011 extended by de-identified data on hospitalizations provided by the Federal Ministry of Health. These data cover approximately 70% of the population of the province of Lower Austria, which is the second largest of the nine Austrian provinces with an average population of 1,605,885 persons between 2008 and 2011 according to Statistics Austria. The remaining 30% of the population are covered by other insurance carriers. The analysis of de-identified health data is conformant with the Austrian law for data protection [14]. In addition to basic demographic descriptors and dates of deaths the data base contains all publicly reimbursed healthcare services in Lower Austria, including medication dispensings coded by the Anatomical Therapeutic Chemical (ATC) classification system [15]. Hospitalization data comprise date of discharge, length of stay and main and associated discharge diagnoses, which are coded by the International Statistical Classification of Diseases and Related Health Problems (ICD-10).

### Study population

**Fig 1:**
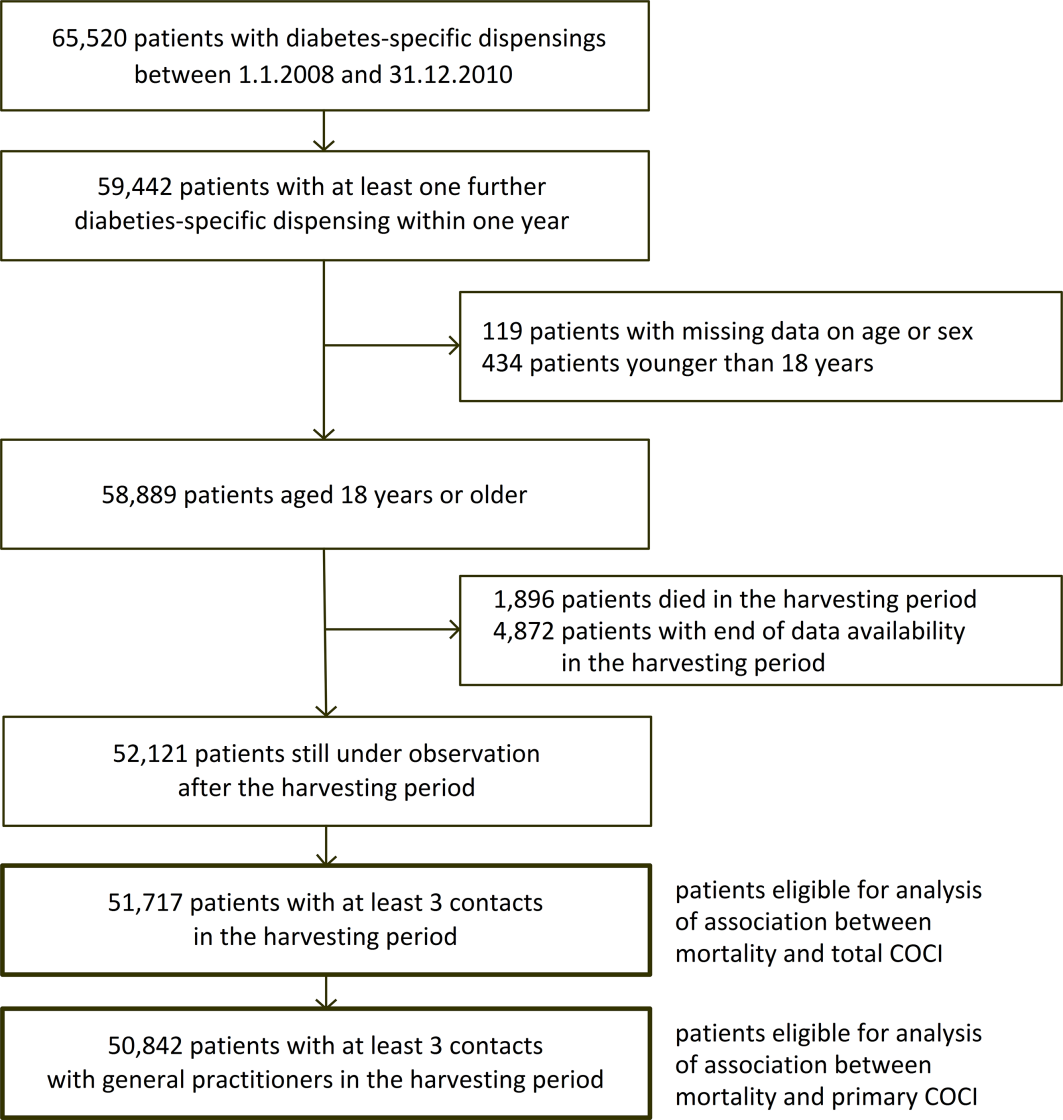
Selection of study patients.

Using the claims data, we identified subjects who received diabetes-specific medicines according to [16] in the years 2008 to 2010. The chronologically first recorded dispensing of any such medicine of each patient is referred to as that patient’s index dispensing. Subjects were eligible for our study if they were 18 years of age or older at the day of their index dispensing and if they had at least one further diabetes-specific dispensing within one year. Since quantifying COC only makes sense for patients who have multiple contacts with healthcare providers, we excluded patients with less than three contacts during the first year after the index dispensing. Subjects were also excluded if their available data records covered less than one year after the date of index dispensing. Fig 1 shows the study population resulting from the described procedure.

### Study design

**Fig 2:**
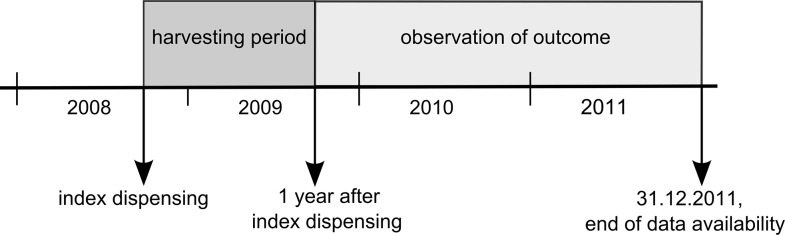
Illustration of study design. For each subject, explanatory variables including the COCI were determined from data available in a ‘harvesting period’ of one year starting with the date of index dispensing. One year after index dispensing was used as starting point for the survival analysis.

In this retrospective cohort study, COCI and other explanatory variables were determined from data available in a ‘harvesting period’ of one year starting with the date of index dispensing. As explanatory variables, we considered age at date of index dispensing, sex, the number of days in hospital and–as proxy for the presence of comorbidities–hospital discharge diagnoses (grouped by ICD-10 chapters) and drug dispensings (grouped by ATC second-level codes). Time from the end of the harvesting period to death or end of data availability, whichever occurred first, was used as the possibly censored outcome variable, see Fig 2. This strict chronological separation between observation of predictors and outcomes mimics the setting of a prospective observational study investigating the association of exposure to high or low COC with mortality.

### Measuring continuity of care

Among the various measures of COC we chose the COCI [12], which is frequently used in literature [17] and was recommended for study populations with many healthcare contacts with potentially many different providers [8]. The COCI assigns a value between 0 and 1 to each patient, with 1 indicating the highest possible COC, i.e. the patient always consults the same healthcare provider. The COCI is defined as

$$\mathrm{COCI}=\frac{\overset{k}{\underset{i=1}{\sum }}n_{i}^{2}-N}{N(N-1)},$$

where *k* is the number of different healthcare providers the patient has seen, *n_i_* denotes the number of contacts with the *i*-th healthcare provider and *N* is the total number of contacts.

For each subject we calculated two types of continuity measures. First, we determined the “total” COC by calculating the COCI from all contacts covered by the public system except for dental care (total COCI). This comprises practitioners from the following disciplines: accident surgery, clinical psychology, CT/MR-institutes, dermatology, general practice, gynaecology, internal medicine, laboratory, logopedics, midwifery, neurology, ophthalmology, orthopaedics, otorhinolaryngology, pneumology, radiology, surgery, and urology. Further, walk-in clinics and institutes as well as hospital stays were included.

**Fig 3:**
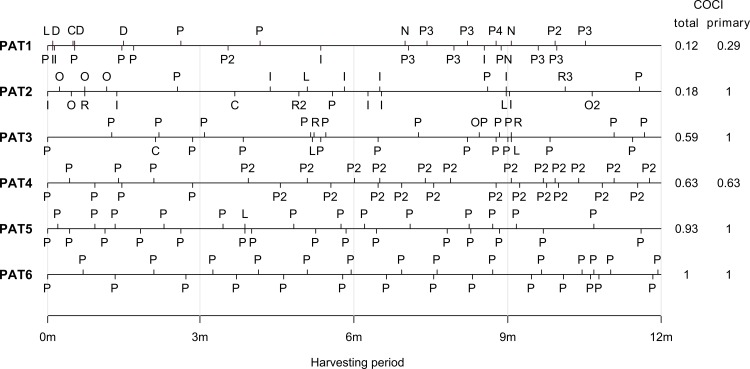
Time course of contacts during the harvesting period exemplarily for six female patients aged between 67 and 71 years. The two columns on the right hand side contain the resulting total and primary COCI values, respectively. Contacts with GPs are marked by “P”, other specialities are coded as follows: C–CT/MR-institutes, D–dermatology, I–internal medicine, L–laboratory, N–neurology, O–orthopedics, R–radiology, OP–ophthalmology, PN–pneumology. Contacts with different healthcare providers of the same specialty are distinguished by consecutive numbers behind the symbol, e.g. patient PAT1 has contacted four different GPs.

Second, we considered only contacts with general practitioners in calculating the COCI, obtaining a measure of continuity of primary care (primary COCI). The total COCI is highly influenced by the diversity of the medical care a patient receives (see Fig 3 where some typical time courses of contacts are depicted). Since the COCI is unstable for patients with few contacts (for *N* = 1 the COCI is not even defined because of division by 0), we only calculated the total COCI for patients with three contacts at any medical disciplines and the primary COCI for patients with three contacts with general practitioners.

For comparison, we also investigated the UPC, given as fraction of contacts with the most frequently seen provider, using either contacts at all medical disciplines covered by the public system except for dental care (“total UPC”) or contacts with general practitioners only (“primary UPC”). Again, the UPC was only determined if there were at least three contacts available.

In contrast to the UPC we are not aware of any simple interpretation of the COCI as, e.g., proportion of contacts. However, as expected, the COCI is larger if care is concentrated among fewer providers. With fixed number of providers *k* and fixed total number of contacts *N*, the COCI is smallest if all providers are seen with the same frequency, i.e. *n*_1_ = … = *n_k_*. The COCI is equal to zero if each provider is seen only once, i.e. *n*_1_ = … = *n_k_* = 1. Further examples of different distributions of contacts among providers and the resulting COCI values were discussed by Steinwachs [10].

### Statistical methods

Categorical variables are described by absolute and relative frequencies, continuous variables by median and interquartile range (IQR). The cumulative incidence of mortality was estimated by the product-limit method [18]. The association between either total or primary COCI and mortality was investigated in univariable and multivariable Cox regression models. The following variables were included in all multivariable models: either *total COCI* or *primary COCI*, *age*, *sex*, the interaction of *age* and *sex*, the *number of contacts*, *hospitalization* (yes/no) and the *length of hospital stays* (days, summed across all hospital stays). Drug dispensings (grouped by ATC second-level codes) and hospital diagnoses (grouped by ICD-10 chapters) concerning at least 1% and not more than 99% of patients were considered as potential additional variables in the model building step, which were selected by backward elimination with Akaike’s information criterion as stopping criterion. While it is common practice to categorize measures of COC before the analysis, we decided to keep them continuous using natural cubic splines. In this way, we avoid problems associated with categorization of continuous variables such as the choice of artificial cut-points or the subsumption of variability within each group [19]. In addition to the explanatory variables *total COCI* and *primary COCI*, *number of contacts* (logarithmized and truncated at the 99^th^ percentile) and *age*, were represented by natural cubic spline bases with 3 degrees of freedom. The variable *length of hospital stay* was logarithmized. Results from Cox models are presented as hazard ratios and 95% confidence intervals. For all continuous variables modelled with splines, we computed hazard ratios between tertile medians (i.e., between the 83.3^th^, the 50^th^ and the 16.7^th^ percentile). Since 61.9% of the patients had a primary COCI of 1, we redefined its comparison values to 1, 0.92 and 0.74, the latter two corresponding to the 28.5^th^ and 9.5^th^ percentiles. A potential time-dependency of the association between COCI and mortality was investigated using Schoenfeld residuals. As sensitivity analysis we repeated the univariable and multivariable Cox regression analysis described above but categorized the total and primary COCI into three groups instead of keeping them continuous using a spline basis. The total COCI was categorized into three equally sized groups using tertiles. In the case of the primary COCI, one group consisted of all subjects having a primary COCI equal to 1 (61.9% of patients) and the other two groups were constructed by splitting the primary COCI at the 19^th^ percentile. Similar univariable and multivariable Cox regression analyses were performed for the primary and total UPC. All statistical analyses were done in R 3.2.2 [20].

## Results

We identified 51,717 patients (“total study population”) eligible for analysis of the association between mortality and the total COCI, see Fig 1. Among the total study population, 50,842 patients (98.3%, “primary study population”) had at least three contacts with GPs in their harvesting periods and were thus considered in the analysis of the association between mortality and the primary COCI. In the following, statements on the total and primary COCI always refer to the respective study populations.

Tables 1 and 2 present patient characteristics for the two study populations overall and by COCI groups. The median age in the total study population was 67 years (IQR: 58, 75) and 53.1% were female. The median total COCI was 0.6 (IQR: 0.43, 0.78) with a median number of 32 contacts (IQR: 22, 46). The majority of patients (31,450; 61.9%) consulted only a single GP, i.e. had a primary COCI equal to 1. Only about one tenth of patients had a primary COCI smaller than 0.75. A higher total COCI was associated with fewer contacts and a lower hospitalization rate. Only 23 out of 50,842 patients in the primary study population had a total COCI higher than the primary COCI.

**Table 1:** Characteristics of the total study population overall and by COCI groups. Cut points for the total COCI groups are tertiles.

	*Total COCI groups*						*Complete study population*	
	*[0*,*0*.*49]*		*(0*.*49*,*0*.*72]*		*(0*.*72*,*1]*			
	N = 17,141		N = 17,471		N = 17,105		N = 51,717	
*Female*	8,923	(52.1%)	9,204	(52.7%)	9,313	(54.4%)	27,440	(53.1%)
*Male*	8,218	(47.9%)	8,267	(47.3%)	7,792	(45.6%)	24,277	(46.9%)
*Age*	66	(57, 72)	67	(58, 74)	68	(60, 77)	67	(58, 75)
*No. of contacts*	36	(26, 49)	32	(23, 45)	27	(18, 41)	32	(22, 46)
*Hospitalization*	4,208	(24.5%)	3,471	(19.9%)	1,729	(10.1%)	9,408	(18.2%)

Sex and Hospitalization are described by N (%), age and the number of contacts by median and quartiles.

**Table 2:** Characteristics of the primary study population, overall and by COCI groups. Since 61.9% of patients had a primary COCI equal to 1, we contrasted a primary COCI equal to 1 with a COCI smaller or greater than the median of COCI-values different from 1, instead of using tertile groups as in Table 1.

	*Primary COCI groups*						*Complete study population*	
	*[0*,*0*.*87]*		*[0*.*87*,*1)*		*[1,1]*			
	N = 10,079		N = 9,313		N = 31,450		N = 50,842	
*Female*	5,445	(54%)	5,168	(55.5%)	16,411	(52.2%)	27,024	(53.2%)
*Male*	4,634	(46%)	4,145	(44.5%)	15,039	(47.8%)	23,818	(46.8%)
*Age*	65	(55, 74)	68	(59, 76)	67	(59, 74)	67	(58, 75)
*No. of contacts*	24	(15, 36)	32	(23, 44)	21	(15, 30)	24	(17, 34)
*Hospitalization*	2,272	(22.5%)	2,346	(25.2%)	4,661	(14.8%)	9,279	(18.3%)

Sex and Hospitalization are described by N (%), age and the number of contacts by median and quartiles.

The median observation time in the total study population was 3.65 years. About one tenth of patients (5,206) died before the end of the study. The cumulative incidence of mortality after one and after two years was 4.2% (95% CI: 4, 4.4) and 8.7% (95% CI: 8.4, 8.9).

**Fig 4:**
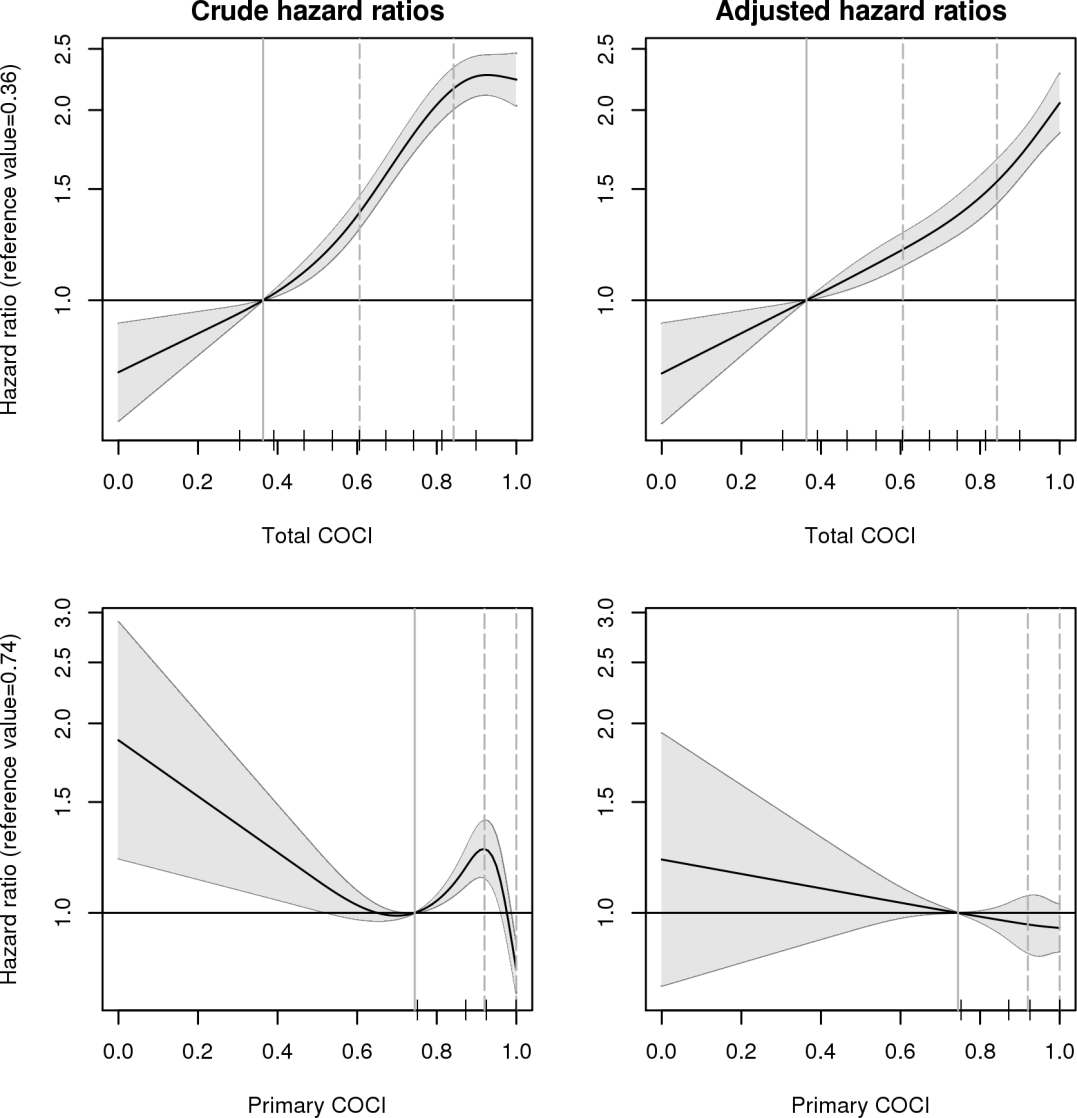
Hazard ratios (mortality) for the total and the primary COCI, with total COCI = 0.36 and primary COCI = 0.74 as reference values and 95% confidence intervals, estimated in univariable and multivariable models. Ticks on the x-axis mark the deciles of the COCI. Solid vertical lines mark the reference values, dashed lines mark comparison values for which hazard ratios are presented in Table 3.

Fig 4, Table 3 and S1 Table present results from univariable and multivariable Cox models investigating the association between all-cause mortality and either total or primary COCI. A higher total COCI was strongly associated with an increased risk of mortality. For instance, the risk of mortality for patients with a total COCI of 0.84 was 2.2-times higher (95% CI: 2.01, 2.33) than for patients with a total COCI of 0.36, estimated in univariable analysis. In multivariable analysis, with the set of adjustment variables comprising age, sex, hospitalization, the number of contacts, drug dispensings and hospital diagnoses, the association between the total COCI and mortality was similar but slightly weaker. In contrast, there was a partially inverse association between the primary COCI and mortality in univariable analysis, yielding a hazard ratio of 0.81 (95% CI: 0.75, 0.88) between primary COCI values of 1 and 0.74. However, the effect was removed after adjusting for covariates (Fig 4 and Table 3). We figured out that adjustment for the number of contacts with GPs was already sufficient to eliminate the association.

**Table 3:** Predictors for mortality. The first column gives the crude hazard ratios (HRs) for the different predictors estimated in univariable Cox regression. The second and third column present the adjusted HRs estimated in the multivariable models containing the total and the primary COCI, respectively. Besides of the variables listed in the table, the model with the total COCI considers 41 ATC-codes and 4 ICD-10-codes. The model with the primary COCI considers 39 ATC-codes and 4 ICD-10-codes. See S1 Table for the adjusted HRs of the ATC-codes and ICD-10-codes. Both multivariable models take into account the interaction between age and sex, thus the estimated effect of age differs between the sexes and the effect of sex differs between different age values, as described in the table.

		*Crude HR (95% CI)*	*Adjusted HR (95% CI)*	
			*Model with total COCI*	*Model with primary COCI*
*Total COCI*	0.36 (T1)	1	1	
	0.61 (T2)	1.38 (1.3, 1.46)	1.2 (1.13, 1.28)	
	0.84 (T3)	2.16 (2.01, 2.33)	1.54 (1.42, 1.67)	
*Primary COCI*	0.74	1		1
	0.92	1.26 (1.14, 1.4)		0.96 (0.86, 1.06)
	1	0.81 (0.75, 0.88)		0.95 (0.87, 1.03)
*Age*	54 (T1)	1		
	67 (T2)	2.15 (1.99, 2.31)		
	78 (T3)	5.59 (5.08, 6.16)		
*Age, females*	54 (T1)		1	1
	67 (T2)		2.01 (1.78, 2.27)	2.07 (1.84, 2.34)
	78 (T3)		4.52 (3.83, 5.32)	4.83 (4.09, 5.69)
*Age, males*	54 (T1)		1	1
	67 (T2)		1.86 (1.68, 2.05)	1.84 (1.67, 2.04)
	78 (T3)		3.63 (3.19, 4.12)	3.66 (3.22, 4.16)
*Sex*	female vs. male	0.98 (0.93, 1.04)		
*Sex, 54-year-olds*	female vs. male		0.53 (0.45, 0.63)	0.51 (0.43, 0.61)
*Sex, 67-year-olds*	female vs. male		0.57 (0.52, 0.63)	0.58 (0.52, 0.63)
*Sex, 78-year-olds*	female vs. male		0.66 (0.61, 0.71)	0.68 (0.62, 0.73)
*Hospitalization*	yes vs. no	1.88 (1.77, 2.01)	1.22 (1.11, 1.35)	1.09 (0.99,1.2)
	length (per doubling)	1.43 (1.39, 1.48)	1.18 (1.14, 1.22)	1.17 (1.13, 1.21)
*No. of contacts with any health care provider*	19 (T1)	1	1	
	32 (T2)	1.41 (1.34, 1.48)	1.08 (1.02, 1.14)	
	53 (T3)	2.26 (2.09, 2.44)	1.12 (1.02, 1.22)	
*No. of contacts with GPs*	14 (T1)	1		1
	24 (T2)	1.51 (1.43, 1.59)		1.15 (1.09, 1.21)
	40 (T3)	2.92 (2.69, 3.17)		1.33 (1.21, 1.46)

T1, T2 and T3 refer to the medians of the first, second and third tertile groups, respectively. Since 61.9% of the patients had a primary COCI of 1, we redefined its comparison values to 0.74, 0.92 and 1, the first two COCI values corresponding to the 9.5th and 28.5th percentiles.

As expected, older age, hospitalizations and a larger number of contacts were associated with an increased risk of mortality, both in univariable and multivariable analyses, see Table 3.

Repeating these analyses but categorizing the total and primary COCI into three groups instead of keeping them continuous yielded similar results. For instance, the adjusted hazard for subjects with a total COCI in the third tertile group (COCI between 0.719 and 1) was estimated to be 1.56-times (95% CI: 1.45, 1.68) higher than for patients in the first tertile group (COCI between 0 and 0.491).

An investigation of the Schoenfeld residuals did not suggest a time-dependency of the association between the COCI and mortality.

The analysis of the relation between the UPC and mortality yielded very similar results to the ones for the COCI, see S2 Table and S1 Fig.

## Discussion

### Findings and possible explanations

In our study population a strong direct association between the total COCI and the risk of mortality was observed, i.e. subjects with higher total COCI were more likely to die earlier. This result does not only stand in contrast with other studies, mostly linking an increased COC to better health outcomes, but is also counter-intuitive: how can an ongoing and sound patient-physician relationship be harmful for a patient? One explanation is that the total COCI, taking into account contacts at all medical disciplines, is inversely related to the degree of multidisciplinarity of care, see Fig 3 for an illustration. This puts a new complexion on our results: it is plausible that patients who benefit from multidisciplinary care, which is reflected by a small total COCI, might have a smaller risk of mortality.

One alternative to the hardly interpretable total COCI is to calculate the COCI from patients' contacts with GPs. The primary COCI and the UPC calculated from contacts with GPs were shown to be inversely related to the risk of mortality [2, 3, 6]. While there was a similar tendency in our study sample, the association was clearly non-significant and with the confidence limits for the adjusted hazard ratios being close to 1, any relevant effect can be ruled out. Shin et al. [5] also state an inverse relation between COCI and mortality, though it was not entirely clear to us whether the COCI was computed based on all visits at any medical discipline or based on visits to GPs only. A study from the United States, focusing on preventable hospitalization, did not find any relation between the total COCI and the risk of mortality [4].

The discrepancy between our results for the total and primary COCI makes clear that it is essential for a sensible interpretation to carefully report which type of medical disciplines had been considered. Nevertheless, Jee and Cabana [8] found in a systematic review on different continuity of care indices deduced from medical records, claims data or surveys, that 13 out of 44 studies did not specify the types of providers.

Aside from the issues of reporting, the interpretability of measures such as the COCI calculated from contacts to different disciplines has to be questioned. One problem is that regardless of whether the care a patient receives is well-orchestrated by a single physician, he will be assigned a low COC if referred to multiple specialists. This was already addressed in the original paper introducing the COCI [12], which suggests to view contacts with referred providers as having been to the referring provider. The underlying assumption seems to be that referring a patient maintains COC even though a new provider is introduced in the care chain (e.g., by information exchange between the two providers). When using administrative data sources for calculating the COCI, however, this idea frequently cannot be applied due to missing referral data. In the way, the total COCI was implemented in our study, it is just a measure of how many different providers a patient has seen regardless of whether these contacts were well coordinated or not. We feel that the primary COCI is better in accordance with the concept of continuity of care. If a patient contacts several GPs, this is reflected by a low primary COCI in concordance with our intuition. On the other hand, it assigns the highest primary COCI to situations, which one also intuitively associates with optimal continuity of care, namely a patient in the care of a single GP.

The before-mentioned aspect may be generalized to another shortcoming of the COCI, namely that it does not consider whether health information is exchanged between the different healthcare providers of a patient. Even though interpersonal COC will suffer when a change of the care provider occurs (even for referrals), exchange of patient information may at least to some extent maintain informational COC. As an example, Shared EHR systems that are currently a fundamental part of the eHealth strategies of most industrial countries could substantially improve informational COC [21]. If we know for example that certain patient information is routinely shared between certain medical disciplines by means of a Shared EHR system, it could make sense to mathematically compensate for this in the calculation of the COCI. In a fully integrated care environment, where all patient information is shared between all care providers and informational COC is thus uniformly high, the COCI may even be seen as reflecting pure interpersonal COC [22].

We received similar results from a sensitivity analysis relating the total and primary UPC to mortality: While a higher total UPC was associated with an increased mortality, there was no relevant association between the primary UPC and mortality. This indicates that the question of deciding which type of medical disciplines to take into account is not only relevant for the COCI but also for other measures of continuity of care.

### Study limitations and strengths

One limitation of the present study is that our database does not contain information on contacts with private healthcare providers or to hospital outpatients’ departments. Assuming that hospital outpatients’ departments are usually contacted for severe or urgent reasons, ignoring these contacts might have led to an overestimation of the observed direct relation between the total COCI and mortality. Of course, the calculation of the primary COCI is independent of the information on these contacts. On the other hand, the omission of contacts with private healthcare providers potentially affects the total as well as the primary COCI, probably leading to an overestimation of these measures. Unfortunately, we could not find any reliable data on the proportion of health care services provided by private physicians. A rough idea can be gained from a telephone survey in summer 2012, which found that every third respondent had contacted a private healthcare provider in the current year [23]. However, we expect the omission of contacts with private providers to have a similar impact on the relation of the total and the primary COCI with mortality, respectively.

Similarly as in most observational studies, the results must be interpreted with caution due to the possibility of unmeasured confounding. In order to control for patients’ morbidity, a potentially strong confounder, we adjusted for the presence of certain hospital diagnoses and drug dispensings.

Further, the COC values of our study population will likely have been influenced by the fact that Austrian patients have a free choice of care providers. They may even directly consult a specialist before having seen a primary care provider. Under these circumstances COC values can be expected to differ from countries with a more rigidly controlled access to care providers [24].

Strengths of our study lie in the population-wide study cohort and in the strict separation of the time period for which the COCI is calculated from the period in which the outcome is observed. In contrast to many other studies, we have not categorized the COCI in the analysis, allowing us to smoothly estimate the risk of mortality over the whole range of COCI values, see Fig 4.

### Implications

Based on our results we strongly advise researchers working with measures of COC to clearly state which types of medical disciplines are taken into account. Moreover, when deciding to use measures based on contacts at all medical disciplines, one should be aware of the difficulties in interpretation. Our study did not show an association between mortality and primary COCI for the diabetic patients in Lower Austria.

## Supporting information

S1 Table Predictors for mortality. ATC-codes and ICD-10-codes selected into the multivariable models with adjusted hazard ratios (HRs, presence vs. absence of the respective drug dispensing or hospital diagnosis). The model with the total COCI considers 41 ATC-codes and 4 ICD-10-codes. The model with the primary COCI considers 39 ATC-codes and 4 ICD-10-codes. See Table 3 for results on the other predictors contained in these models.
(PDF)

S2 Table Predictors for mortality. The first column gives the crude hazard ratios (HRs) for the different predictors estimated in univariable Cox regression. The second and third column present the adjusted HRs estimated in the multivariable models containing the total and the primary UPC, respectively. Besides of the variables listed in the table, the model with the total UPC considers 41 ATC-codes and 4 ICD-10-codes. The model with the primary UPC considers 39 ATC-codes and 4 ICD-10-codes. Both multivariable models take into account the interaction between age and sex, thus the estimated effect of age differs between the sexes and the effect of sex differs between different age values, as described in the table.
(PDF)

S1 Fig Hazard ratios (mortality) for the total and the primary UPC, with total UPC = 0.56 and primary UPC = 0.86 as reference values and 95% confidence intervals estimated in univariable and multivariable models. Ticks on the x-axis mark the deciles of the UPC. Solid vertical lines mark the reference values, dashed lines mark comparison values for which hazard ratios are presented in S2 Table.
(TIFF)
