# Supplementary material for: Mortality and continuity of care – Definitions matter! A cohort study in diabetics
Source: PLoS One. 2018 Jan 19;13(1):e0191386. doi: 10.1371/journal.pone.0191386 (PMC5774784; doi:10.1371/journal.pone.0191386)
Supplement: S1 Table — ATC-codes and ICD-10-codes selected into the multivariable models with adjusted hazard ratios (HRs, presence vs. absence of the respective drug dispensing or hospital diagnosis). The model with the total COCI considers 41 ATC-codes and 4 ICD-10-codes. The model with the primary COCI considers 39 ATC-codes and 4 ICD-10-codes. See Table 3 for results on the other predictors contained in these models. (PDF) [file pone.0191386.s001.pdf]

S1 Table. ***Predictors for mortality.*** *ATC-codes and ICD-10-codes selected into the multivariable models with adjusted hazard ratios (HRs, presence vs. absence of the respective drug dispensing or hospital diagnosis). The model with the total COCI considers 41 ATC-codes and 4 ICD-10-codes. The model with the primary COCI considers 39 ATC-codes and 4 ICD-10-codes. See Table 3 for results on the other predictors contained in these models.*

		Adjusted HR (95% CI)	
		Model with total COCI	*Model with primary COCI*
A02	*DRUGS FOR ACID RELATED DISORDERS*	1.11 (1.04, 1.19)	1.08 (1.02, 1.16)
A05	*BILE AND LIVER THERAPY*	1.29 (1.13, 1.47)	1.28 (1.12, 1.46)
A06	*DRUGS FOR CONSTIPATION*	1.19 (1.1, 1.28)	1.18 (1.09, 1.28)
A07	*ANTIDIARRHEALS, INTESTINAL ANTIINFLAMMATORY/ANTIINFECTIVE AGENTS*	1.09 (1.01, 1.18)	1.08 (1, 1.16)
A11	*VITAMINS*	0.91 (0.84, 0.99)	0.9 (0.82, 0.98)
B01	*ANTITHROMBOTIC AGENTS*	1.2 (1.13, 1.28)	1.19 (1.12, 1.27)
B03	*ANTIANEMIC PREPARATIONS*	1.35 (1.24, 1.47)	1.35 (1.24, 1.47)
C01	*CARDIAC THERAPY*	1.21 (1.14, 1.29)	1.2 (1.13, 1.28)
C03	*DIURETICS*	1.64 (1.54, 1.75)	1.64 (1.54, 1.75)
C04	*PERIPHERAL VASODILATORS*	1.07 (0.98, 1.15)	1.06 (0.98, 1.15)
C09	*AGENTS ACTING ON THE RENIN-ANGIOTENSIN SYSTEM*	0.86 (0.81, 0.92)	0.85 (0.8, 0.9)
C10	*LIPID MODIFYING AGENTS*	0.74 (0.7, 0.78)	0.72 (0.68, 0.76)
D01	*ANTIFUNGALS FOR DERMATOLOGICAL USE*	0.9 (0.83, 0.99)	0.9 (0.83, 0.98)
D03	*PREPARATIONS FOR TREATMENT OF WOUNDS AND ULCERS*	1.27 (1.15, 1.4)	1.27 (1.15, 1.4)
D06	*ANTIBIOTICS AND CHEMOTHERAPEUTICS FOR DERMATOLOGICAL USE*	1.08 (0.97, 1.2)	not selected
D08	*ANTISEPTICS AND DISINFECTANTS*	1.21 (1.1, 1.32)	1.22 (1.11, 1.33)
D11	*OTHER DERMATOLOGICAL PREPARATIONS*	1.1 (0.97, 1.25)	not selected
G03	*SEX HORMONES AND MODULATORS OF THE GENITAL SYSTEM*	0.67 (0.56, 0.79)	0.63 (0.53, 0.75)
G04	*UROLOGICALS*	0.92 (0.86, 0.99)	0.89 (0.83, 0.96)
H02	*CORTICOSTEROIDS FOR SYSTEMIC USE*	1.13 (1.04, 1.24)	1.08 (0.99, 1.19)
H03	*THYROID THERAPY*	0.88 (0.81, 0.96)	0.88 (0.81, 0.96)
L01	*ANTINEOPLASTIC AGENTS*	1.94 (1.63, 2.31)	1.89 (1.58, 2.25)
L02	*ENDOCRINE THERAPY*	1.25 (1.08, 1.45)	1.17 (1.01, 1.36)
L03	*IMMUNOSTIMULANTS*	2.4 (1.98, 2.91)	2.31 (1.9, 2.81)
M01	*ANTIINFLAMMATORY AND ANTIRHEUMATIC PRODUCTS*	0.82 (0.77, 0.86)	0.8 (0.76, 0.85)
M02	*TOPICAL PRODUCTS FOR JOINT AND MUSCULAR PAIN*	0.89 (0.82, 0.96)	0.91 (0.84, 0.98)
M03	*MUSCLE RELAXANTS*	0.9 (0.8, 1.01)	0.89 (0.79, 1)
M04	*ANTIGOUT PREPARATIONS*	1.09 (1.02, 1.17)	1.07 (1, 1.15)

M05	*DRUGS FOR TREATMENT OF BONE DISEASES*	0.91 (0.82, 1.01)	0.89 (0.8, 0.99)
N01	*ANESTHETICS*	0.76 (0.67, 0.88)	0.74 (0.65, 0.85)
N02	*ANALGESICS*	1.23 (1.15, 1.32)	1.24 (1.16, 1.32)
N03	*ANTIEPILEPTICS*	1.21 (1.1, 1.33)	1.17 (1.06, 1.29)
N04	*ANTI-PARKINSON DRUGS*	1.08 (0.97, 1.2)	not selected
N05	*PSYCHOLEPTICS*	1.33 (1.24, 1.42)	1.34 (1.26, 1.44)
N06	*PSYCHOANALEPTICS*	1.15 (1.08, 1.22)	1.13 (1.06, 1.2)
N07	*OTHER NERVOUS SYSTEM DRUGS*	0.82 (0.72, 0.93)	0.81 (0.72, 0.92)
R01	*NASAL PREPARATIONS*	0.75 (0.66, 0.86)	0.75 (0.66, 0.86)
R03	*DRUGS FOR OBSTRUCTIVE AIRWAY DISEASES*	1.21 (1.13, 1.3)	1.18 (1.1, 1.27)
R05	*COUGH AND COLD PREPARATIONS*	1.08 (1, 1.16)	1.09 (1.01, 1.17)
S01	*OPHTHALMOLOGICALS*	not selected	0.91 (0.85, 0.98)
S02	*OTOLOGICALS*	0.76 (0.59, 0.97)	0.76 (0.59, 0.97)
V03	*ALL OTHER THERAPEUTIC PRODUCTS*	1.45 (1.24, 1.7)	1.46 (1.25, 1.71)
Chapter 2	*NEOPLASMS*	1.68 (1.49, 1.9)	1.67 (1.47, 1.88)
Chapter 4	*ENDOCRINE, NUTRITIONAL AND METABOLIC DISEASES*	1.19 (1.07, 1.32)	1.19 (1.07, 1.32)
Chapter 5	*MENTAL AND BEHAVIOURAL DISORDERS*	1.29 (1.12, 1.49)	1.29 (1.12, 1.48)
Chapter 13	*DISEASES OF THE MUSCULOSKELETAL SYSTEM AND CONNECTIVE TISSUE*	0.71 (0.62, 0.82)	0.71 (0.62, 0.82)
