# Supplementary material for: Mortality and continuity of care – Definitions matter! A cohort study in diabetics
Source: PLoS One. 2018 Jan 19;13(1):e0191386. doi: 10.1371/journal.pone.0191386 (PMC5774784; doi:10.1371/journal.pone.0191386)
Supplement: S2 Table — The first column gives the crude hazard ratios (HRs) for the different predictors estimated in univariable Cox regression. The second and third column present the adjusted HRs estimated in the multivariable models containing the total and the primary UPC, respectively. Besides of the variables listed in the table, the model with the total UPC considers 41 ATC-codes and 4 ICD-10-codes. The model with the primary UPC considers 39 ATC-codes and 4 ICD-10-codes. Both multivariable models take into account the interaction between age and sex, thus the estimated effect of age differs between the sexes and the effect of sex differs between different age values, as described in the table. (PDF) [file pone.0191386.s002.pdf]

S2 Table. ***Predictors for mortality.*** *The first column gives the crude hazard ratios (HRs) for the different predictors estimated in univariable Cox regression. The second and third column present the adjusted HRs estimated in the multivariable models containing the total and the primary UPC, respectively. Besides of the variables listed in the table, the model with the total UPC considers 41 ATC-codes and 4 ICD-10-codes. The model with the primary UPC considers 39 ATC-codes and 4 ICD-10-codes. Both multivariable models take into account the interaction between age and sex, thus the estimated effect of age differs between the sexes and the effect of sex differs between different age values, as described in the table.*

		Crude HR (95% CI)	*Adjusted HR (95% CI)*	
			Model with total UPC	*Model with primary UPC*
Total UPC	0.56 (T1)	1	1	
	0.78 (T2)	1.39 (1.33, 1.46)	1.17 (1.12, 1.24)	
	0.92 (T3)	2.09 (1.93, 2.26)	1.52 (1.41, 1.65)	
Primary UPC	0.86	1		1
	0.96	1.16 (1.05, 1.28)		0.96 (0.88, 1.06)
	1	0.78 (0.72, 0.84)		0.95 (0.87, 1.03)
Age	54 (T1)	1		
	67 (T2)	2.15 (1.99, 2.31)		
	78 (T3)	5.59 (5.08, 6.16)		
Age, females	54 (T1)		1	1
	67 (T2)		2.02 (1.79, 2.28)	2.07 (1.83, 2.34)
	78 (T3)		4.54 (3.85, 5.35)	4.83 (4.09, 5.69)
Age, males	54 (T1)		1	1
	67 (T2)		1.85 (1.68, 2.05)	1.84 (1.67, 2.04)
	78 (T3)		3.63 (3.20, 4.13)	3.66 (3.22, 4.16)
Sex	female vs. male	0.98 (0.93, 1.04)		
Sex, 54-year-olds	female vs. male		0.53 (0.44, 0.63)	0.51 (0.43, 0.61)
Sex, 67-year-olds	female vs. male		0.57 (0.52, 0.63)	0.58 (0.52, 0.63)
Sex, 78-year-olds	female vs. male		0.66 (0.61, 0.71)	0.68 (0.62, 0.73)
Hospitalization	yes vs. no	1.88 (1.77, 2.01)	1.22 (1.1, 1.34)	1.09 (0.99, 1.2)
	length (per doubling)	1.43 (1.39, 1.48)	1.27 (1.21, 1.34)	1.25 (1.19, 1.32)
No. of contacts with any health care provider	19 (T1)	1	1	
	32 (T2)	1.41 (1.34, 1.48)	1.08 (1.02, 1.14)	
	53 (T3)	2.26 (2.09, 2.44)	1.12 (1.03, 1.22)	
No. of contacts with GPs	14 (T1)	1		1
	24 (T2)	1.51 (1.43, 1.59)		1.15 (1.08, 1.21)
	40 (T3)	2.92 (2.69, 3.17)		1.33 (1.21, 1.46)

T1, T2 and T3 refer to the medians of the first, second and third tertile groups, respectively. Since 61.9% of the patients had a primary UPC of 1, we redefined its comparison values to 0.86, 0.96 and 1, the first two UPC values corresponding to the 9.5th and 28.5th percentiles.
