# Supplementary figures and images for: Mortality and continuity of care – Definitions matter! A cohort study in diabetics
Source: PLoS One. 2018 Jan 19;13(1):e0191386. doi: 10.1371/journal.pone.0191386 (PMC5774784; doi:10.1371/journal.pone.0191386)

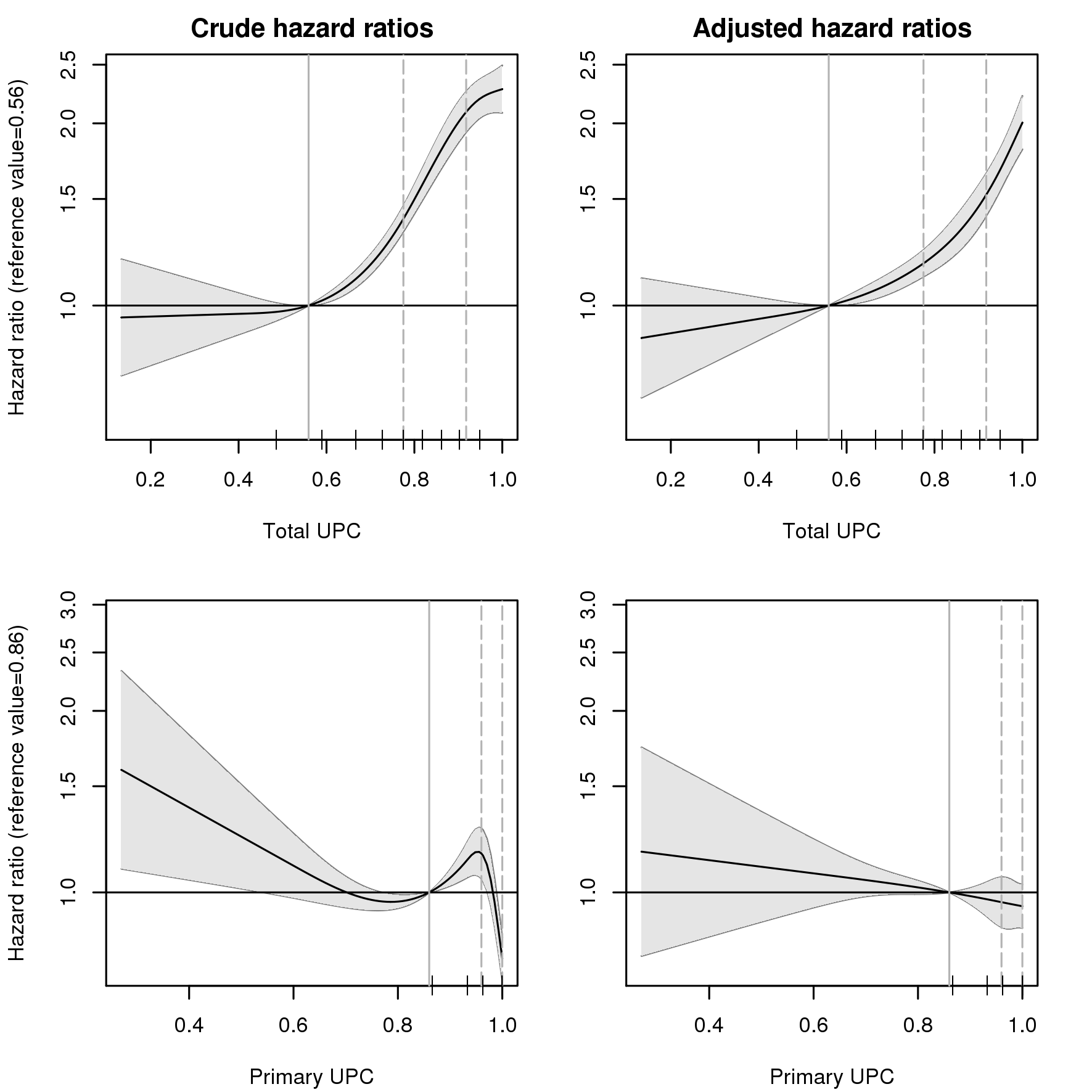

Supplement: S1 Fig — Ticks on the x-axis mark the deciles of the UPC. Solid vertical lines mark the reference values, dashed lines mark comparison values for which hazard ratios are presented in S2 Table. (TIFF) [file pone.0191386.s003.tiff]
